# Melatonin and omega-3 neuroprotection in prenatal rat spinal cord exposed to 900 MHz electromagnetic field

**DOI:** 10.17305/bb.2025.12633

**Published:** 2025-07-21

**Authors:** Ömür Gülsüm Deniz, Gamze Altun, Süleyman Kaplan

**Affiliations:** 1 Department of Histology and Embryology, Faculty of Medicine, Bolu Abant İzzet Baysal University, Bolu, Türkiye; 2 Department of Histology and Embryology, Faculty of Medicine, Ondokuz Mayıs University, Samsun, Türkiye

**Keywords:** Electromagnetic field, neuroprotection, physical disector, Cavalieri principle, ultrastructure, spinal cord

## Abstract

The electromagnetic field (EMF) emitted by electronic devices induces pathological changes in tissues, adversely affecting embryonic and pubertal development. This study investigates the effects of melatonin (Mel) and omega-3 fatty acids (ω3) on the spinal cord in rats exposed to EMF, employing stereological methods alongside light and electron microscopic evaluations. Pregnant Wistar albino rats were divided into seven groups: Control (CONT), sham-exposed (SHAM), EMF alone, EMF-Mel, EMF-ω3, Mel only, and ω3 only. The EMF, EMF-Mel, and EMF-ω3 groups were exposed to a 900 MHz EMF for two hours daily during the prenatal period (21 days). Mel and ω3 were administered via intragastric gavage prior to EMF exposure. Upon completion of the experiment (on the 35th day post-birth), spinal cord tissues of all male offspring were dissected and subjected to light and ultrastructural examinations. Stereological analyses calculated grey matter (GM) to total volume ratios, white matter (WM) to total volume ratios, GM to WM volume ratios, total spinal cord volume, and motor neuron counts. No significant differences were observed among the groups regarding GM/WM volume ratios, GM/total volume ratios, WM/total volume ratios, and total spinal cord volume (*P* > 0.05). However, a significant reduction in motor neuron numbers was noted in the EMF-ω3 group compared to the CONT group (*P* < 0.01). Light and ultrastructural examinations revealed marked motor neuron degeneration and axonal disruption in the EMF group, which were mitigated in the Mel and ω3-treated groups. These findings indicate that prenatal exposure to 900 MHz EMF exerts detrimental effects on spinal cord tissue and underscore the necessity for further studies exploring varying doses and durations to elucidate the potential effects of ω3 and Mel.

## Introduction

The rapid advancement of technology is significant not only for the benefits it offers but also for the individual and societal side effects it may induce. The proliferation of technological devices in daily life is accelerating, particularly those that generate electromagnetic fields (EMFs). Research indicates that exposure to increasing levels of EMF can lead to pathological outcomes, underscoring the necessity of investigating the effects of EMF exposure on human health [1]. Among the primary sources of EMF that impact individual health are mobile phones, which have become essential tools, alongside the transmitters that facilitate communication. This is particularly concerning, as mobile phones are the EMF sources closest to the human body. The EMF emitted by these devices can significantly affect living tissue, with effects manifesting differently across various tissues and organs [2]. Numerous studies have documented that specific levels of EMF can induce morphological, biochemical, and physiological changes in organs such as the testes, brain, ovaries, liver, kidneys, and spinal cord, potentially leading to reversible or irreversible pathologies [3–8]. Depending on the intensity and duration of exposure, EMF has been shown to produce particularly harmful effects on the central nervous system [6, 9, 10].

Antioxidants play a crucial role in protecting against oxidative damage caused by free radicals. A balance between free radicals and antioxidants is essential for maintaining vital biological functions. Melatonin (Mel) is one of the most potent antioxidants available today. Due to its hydrophilic properties, Mel readily crosses the blood-brain barrier and diffuses quickly into various subcellular compartments [11, 12]. Beyond its antioxidant capabilities, Mel exhibits numerous bioactivities, including anti-inflammatory, anticarcinogenic, neuroprotective, and anti-aging effects. Notably, Mel acts as a neuroprotective agent with receptors located in the fetal brain [13], demonstrating a dose-dependent effect against brain damage [14]. Consequently, Mel plays a critical role in fetal development [15].

Omega-3 (ω3) fatty acids are unsaturated fats with notable antioxidant properties that cannot be synthesized by the body and must be obtained through diet [16]. These fatty acids are integral to cellular membrane structure and are essential for maintaining normal cellular functions. They also possess antioxidant, anti-inflammatory, antihypertensive, antihyperlipidemic, and antiatherogenic properties. Omega-3 fatty acids are particularly abundant in the brain, spinal cord, retina, and other neural tissues, contributing significantly to nervous system development. They facilitate the healthy transmission of electrical impulses by protecting axonal structures [17].

Pathological changes resulting from spinal cord tissue damage can disrupt signal transmission between the brain and the peripheral nervous system [9]. Our review of the current literature identified a scarcity of studies investigating the effects of 900 MHz EMF on the spinal cord. Therefore, this study aims to explore the effects of Mel and ω3 on the spinal cords of male pups exposed to 900 MHz EMF during the prenatal period. Utilizing stereological methods—an unbiased and reliable histomorphometric approach—as well as light and electron microscopic evaluations, this research seeks to contribute valuable insights to the existing literature.

## Materials and methods

### Animals and group design

The spinal cords of animals used in this study were obtained in accordance with ethical guidelines approved by the Ondokuz Mayıs University Animal Experiments Local Ethics Committee (Approval No. 68489742–604.01.03-E.5621, Date: 07.03.2019). The spinal cords were dissected following perfusion and served as the tissue for the study (Study No. 2018/24, Date: 05-04-2018). The welfare of the experimental animals and adherence to ethical principles were prioritized throughout the experiment. All procedures complied with the U.K. Animals (Scientific Procedures) Act 1986, EU Directive 2010/63/EU for animal experiments, and the National Institutes of Health Guidelines for the Care and Use of Laboratory Animals (NIH Publications No. 8023, revised 1978).

A total of thirty-five adult female *Wistar albino* rats were randomly assigned into seven groups, each consisting of five animals. These rats were mated with male rats in separate cages. Rats exhibiting sperm in vaginal smear samples were designated as being on day 0 of pregnancy. Pregnant rats were housed individually at room temperature with 40%–50% humidity, under a 12-h light/dark cycle, and were provided ad libitum access to water and standard chow throughout the experiment.

A power analysis test was conducted using Minitab version 18.0 to determine the requisite number of animals in all groups. In this context, pregnant rats were divided into seven groups, Control (CONT), SHAM, 900 MHz EMF exposure (EMF), a group receiving Mel before 900 MHz EMF exposure (EMF-Mel), a group receiving ω3 prior to 900 MHz EMF exposure (EMF-ω3), a group receiving Mel only (Mel) and one receiving ω3 only (ω3). Female rats in the SHAM group were placed in the EMF exposure system during the gestational period (21 days) without actual EMF treatment. Pregnant rats in the EMF, EMF-Mel, and EMF-ω3 groups were exposed to 900 MHz EMF throughout gestation. The EMF-Mel and EMF-ω3 groups received 50 mg/kg/day of Mel (Lyophilized Melatonin Powder M5250, Sigma-Aldrich, Germany) and 1 mL/kg/day of ω3 (F8020, Sigma-Aldrich, Germany), respectively, via intragastric gavage prior to EMF exposure. A ready-to-use solution of ω3 (Sigma-Aldrich, Germany) at a concentration of 0.93 g/mL was administered.

Female rats that had mated prior to the experiment were randomly divided into groups. With five pregnant rats in each group, at least 10 male newborn rats were obtained from each group by the end of the pregnancy. These male offspring remained with their mothers after birth. Male offspring born to mothers in each group (*n* ═ 5) were randomly selected. Postnatally, male offspring rats (*n* ═ 10) were housed in separate cages after weaning and were sacrificed 35 days post-birth.

### EMF exposure system

Pregnant rats from the EMF exposure groups were housed in an EMF exposure system with a 0–9 W output signal generator (Microwave Test Transmitter, Set Electronic Ltd, Türkiye) emitting 900 MHz radiofrequency (RF)-EMF waves for 2 h a day for 21 days. RF waves were transmitted via a monopole antenna positioned 3.6 cm from the rats’ head region. During exposure, the rats were placed in a Plexiglas apparatus consisting of 16 equal chambers, with ventilation holes in the top to facilitate breathing. The electric field strength was measured using an RF-EMF strength meter (Extech Instruments Corporation, USA), with measurements taken from the caudal region recorded every six minutes in volts per meter (V/m). The EMF exposure experiments were conducted in a soundproof room designed to minimize external interference.

### Paraffin blocks of spinal cord tissue

Thirty-five days postnatally, seventy male rats underwent intracardiac perfusion under anesthesia, utilizing a mixture of ketamine (50 mg/kg) and xylazine (10 mg/kg) administered intraperitoneally. To ensure complete clearance of blood from the vessels, 0.9% physiological saline was perfused intracardially. Following this, a 10% formalin solution was perfused for three to four minutes. Spinal cord tissues were subsequently dissected at the C3-C5 vertebral level. The tissue samples were fixed in a 10% formaldehyde solution to create paraffin blocks and in 4% glutaraldehyde for resin blocks. Tissues that achieved complete fixation underwent routine processing and were embedded in paraffin blocks.

Sections were obtained from the paraffin blocks according to a systematic sampling method, which is an unbiased and efficient approach for selecting sample sites within the region of interest. The sections were stained with cresyl violet (C5042-10 g, Sigma-Aldrich, Germany) and subjected to histopathological evaluations and stereological analyses. Related images were captured using a light microscope compatible with the computer-aided cellSens Entry software (Olympus, Center Valley, PA, USA). During the paraffin embedding process, all samples were coded to ensure that subsequent analyses were conducted blindly.

### Sectioning and stereological analysis

**Figure 1.**
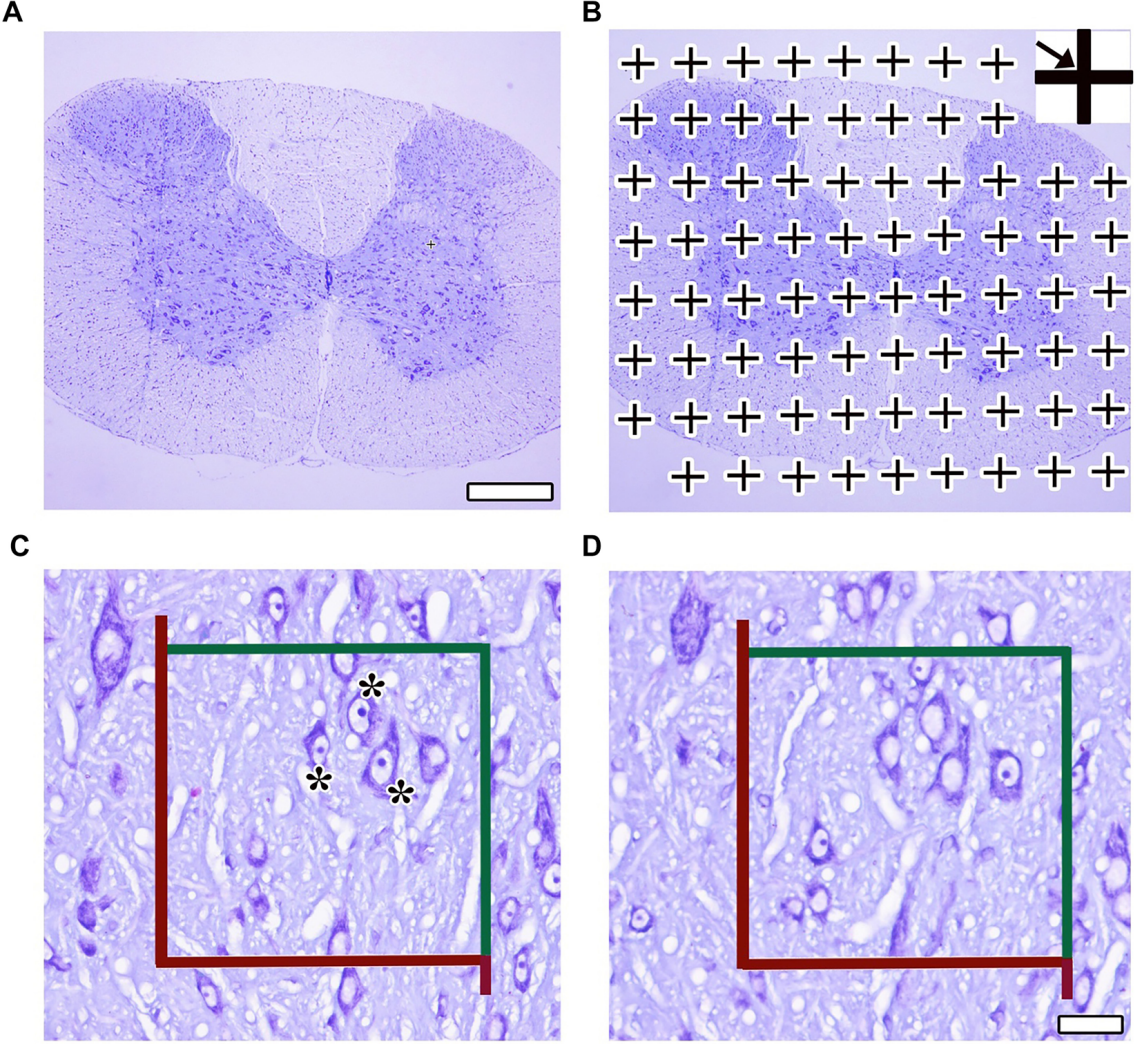
**Representative micrographs showing the stereological application of the Cavalieri principle (A and B) and the physical dissector (C and D).** (A) Panoramic image of the spinal cord; (B) A dotted area ruler superimposed over the panoramic image to apply the Cavalieri principle; (C and D) Consecutive serial sections taken to apply the physical disector: (C) reference section; (D) look-up section. Red lines within the counting frame show the forbidden edges, while green lines show the included edges. (*): These indicate the disector particles, nucleoli that are seen in reference section but not in look up section, can be counted according to the counting frame and the physical disector rules. Original magnification: ×100.

Following the pilot study, 4 µm thick sections were obtained from paraffin-embedded spinal cord tissues at a ratio of 1:15, selecting every 15th section and its adjacent section to form disector pairs in the coronal plane, in accordance with systematic random sampling rules for stereological analyses. After deparaffinization, the physical disector method was employed to estimate the number of motor neurons in the cresyl violet-stained sections (Figure 1C and 1D). The counting unit in this study was defined as the neuronal nucleus, and the area of the unbiased counting frame used was 5925 µm^2^. The total volume of spinal cord samples was calculated using the Cavalieri method [18] (Figure 1A and 1B). In addition, ratios of grey matter (GM)/total volume, white matter (WM)/total volume, and GM/WM volume were estimated using a point counting grid (one point area: 2500 µm^2^). The appropriate coefficient of error (CE ≤ 0.05) for each subject and the appropriate coefficient of variation (CV ≤ 0.20) for each group were considered during stereological estimations [18, 19].

### Preparation of resin blocks for light and electron microscopy

Following perfusion, the spinal cord tissues fixed in 4% glutaraldehyde solution were dissected in a 1 mm^3^ volume under a stereomicroscope, and an electron microscopic tissue tracking procedure was applied [19]. In this context, tissues were treated with phosphate buffer (0.1 M PBS) before processing. Post-fixation was achieved by incubating the tissues in 1% osmium tetroxide (Sigma, St. Louis, MO, USA). Subsequently, the tissues were dehydrated by passing them through graded acetones (50%, 70%, 96%, and 100%) and then transferred to propylene oxide (Sigma-Aldrich Co.). Following this, the tissues were placed in a mixture of araldite (CY212, Agar Scientific Ltd, Essex, UK) and propylene oxide, before being transferred to pure araldite. The spinal cord tissues were embedded in resin molds at 45 ^∘^C. The resin molds were dried overnight and then placed in an oven at 50 ^∘^C, with temperature adjustments made every 30 min. The polymerization process, involving a temperature increase to 50 ^∘^C and then 55 ^∘^C, was completed at 62 ^∘^C for 48 h. The resulting tissue resin blocks were then prepared for sectioning using an ultramicrotome.

### Toluidine blue staining and transmission electron microscopy

Semi-thin sections (500 nm) for light microscopic evaluation and thin sections (70 nm) for electron microscopic evaluation were prepared from resin-embedded tissues. Semi-thin sections were stained with a 1% toluidine blue solution, composed of 1% toluidine blue, 2% sodium borate, and 10 mL distilled water (Sigma-Aldrich Co. LLC., St. Louis, USA). Thin sections were mounted on copper grids and stained using an automatic contrast system (Leica EM AC20, Leica Microsystems GmbH, Germany) with 0.5% uranyl acetate and 3% lead citrate. Ultrastructural images were obtained using electron microscopy (JEOL JSM-7001F, JEOL Ltd., Tokyo, Japan). Electron microscopic examinations were conducted at the Imaging and Characterization Unit of the Ondokuz Mayıs University Karadeniz Advanced Technical Research and Application Center.

### Ethical statement

This study received approval from the Ondokuz Mayıs University Animal Experiments Local Ethics Committee (Approval No. 68489742-604.01.03-E.5621) on March 7, 2019. All experimental procedures were conducted in accordance with the U.K. Animals (Scientific Procedures) Act 1986, the EU Directive 2010/63/EU on animal experimentation, and the National Institutes of Health’s Guide for the Care and Use of Laboratory Animals (NIH Publications No. 8023, revised 1978).

### Statistical analysis

Statistical analyses were performed using SPSS version 21.0 software. The Shapiro–Wilk test assessed the adherence of the data to the normal distribution assumption. For the comparison of continuous variables, normally distributed data were analyzed using One-Way ANOVA, with the Bonferroni test employed for post-hoc comparisons. For non-normally distributed data among multiple groups, the Kruskal–Wallis test was conducted, followed by the Bonferroni-corrected Mann–Whitney *U* test for post-hoc analysis. A significance level of *P* < 0.05 was considered statistically significant.

## Results

### Histopathological analysis

#### Light microscopic evaluation of cresyl violet stained sections

**Figure 2.**
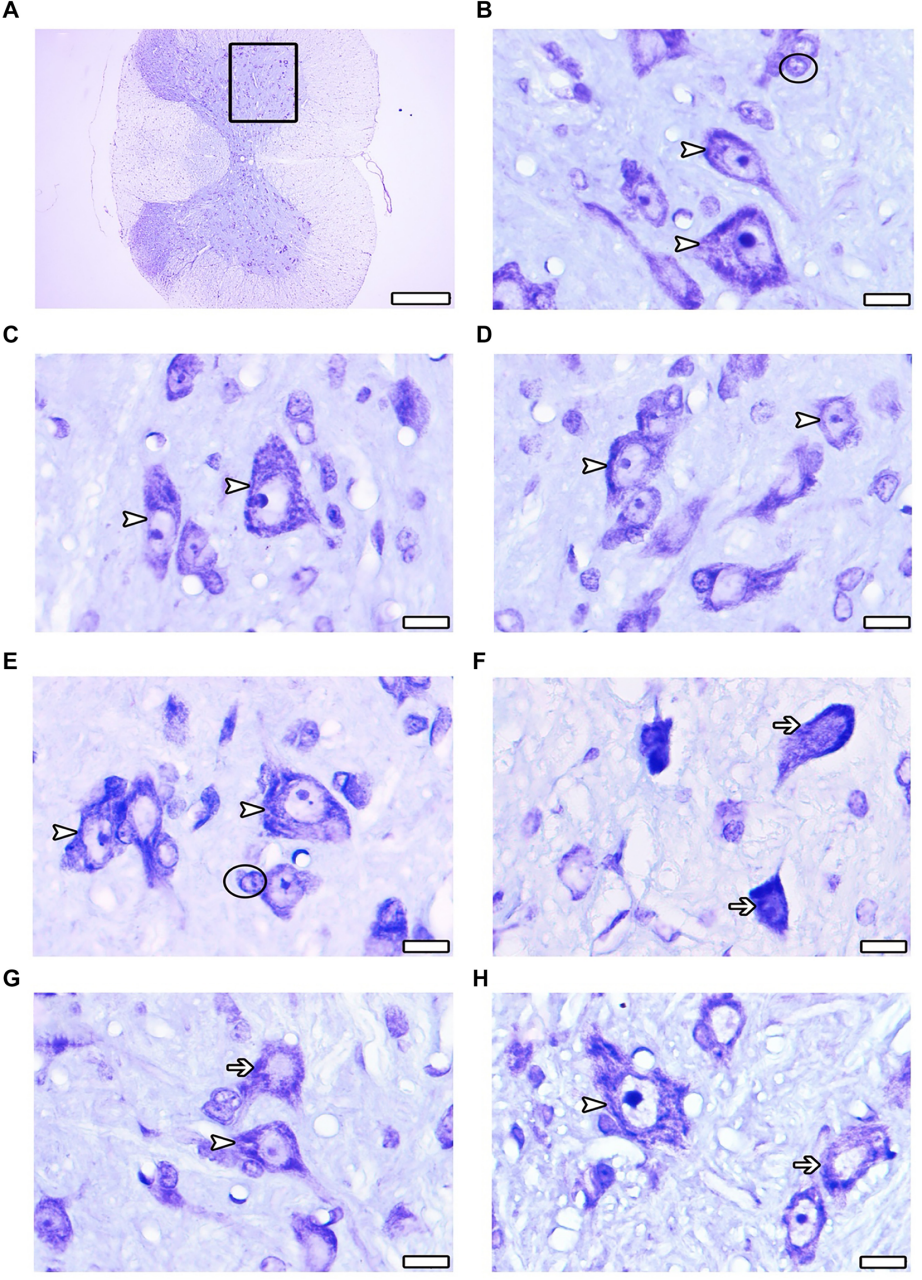
**(A–H) Cresyl violet-stained spinal cord sections after 2-h exposure to EMF.** (A) Panoramic view of the spinal cord; (B) CONT group; (C) SHAM group; (D) Mel group; (E) ω3 group; (F) EMF group; (G) EMF-Mel group; (H) EMF-ω3 group. Arrowhead: Healthy motor neurons; Arrow: Degenerated motor neurons; Circle: Glia cell. Original magnification: ×100.

Cresyl violet-stained sections from all groups were examined histopathologically at the light microscopic level. The histopathological examination of the study tissue sections revealed a generally normal structure in the spinal cord and motor neurons across the CONT (Figure 2B), SHAM (Figure 2C), Mel (Figure 2D), and ω3 (Figure 2E) groups. Light microscopic analysis indicated that the boundaries of the perikarya of motor neurons were clear and distinct. Sugimoto et al. [20] reported that damaged neurons exhibit three main characteristics: irregular cellular outlines and increased chromatin density in both the nucleus and cytoplasm (Figure 2F). In the EMF group (Figure 2F), numerous nuclei were observed that had lost their euchromatic properties, alongside motor neurons with narrow, dark-stained cytoplasm. Most motor neurons in this group displayed indistinct cell boundaries, with the borders of degenerating neurons being particularly hard to identify. In some instances, the cell borders of these damaged neurons were undetectable. Additionally, healthy-appearing motor neurons in the EMF-Mel (Figure 2G) and EMF-ω3 (Figure 2H) groups were more prevalent than in the EMF group. Moreover, degeneration in the EMF-ω3 group was more pronounced than that in the EMF-Mel group.

#### Light microscopic evaluation of toluidine blue stained semi-thin sections

**Figure 3.**
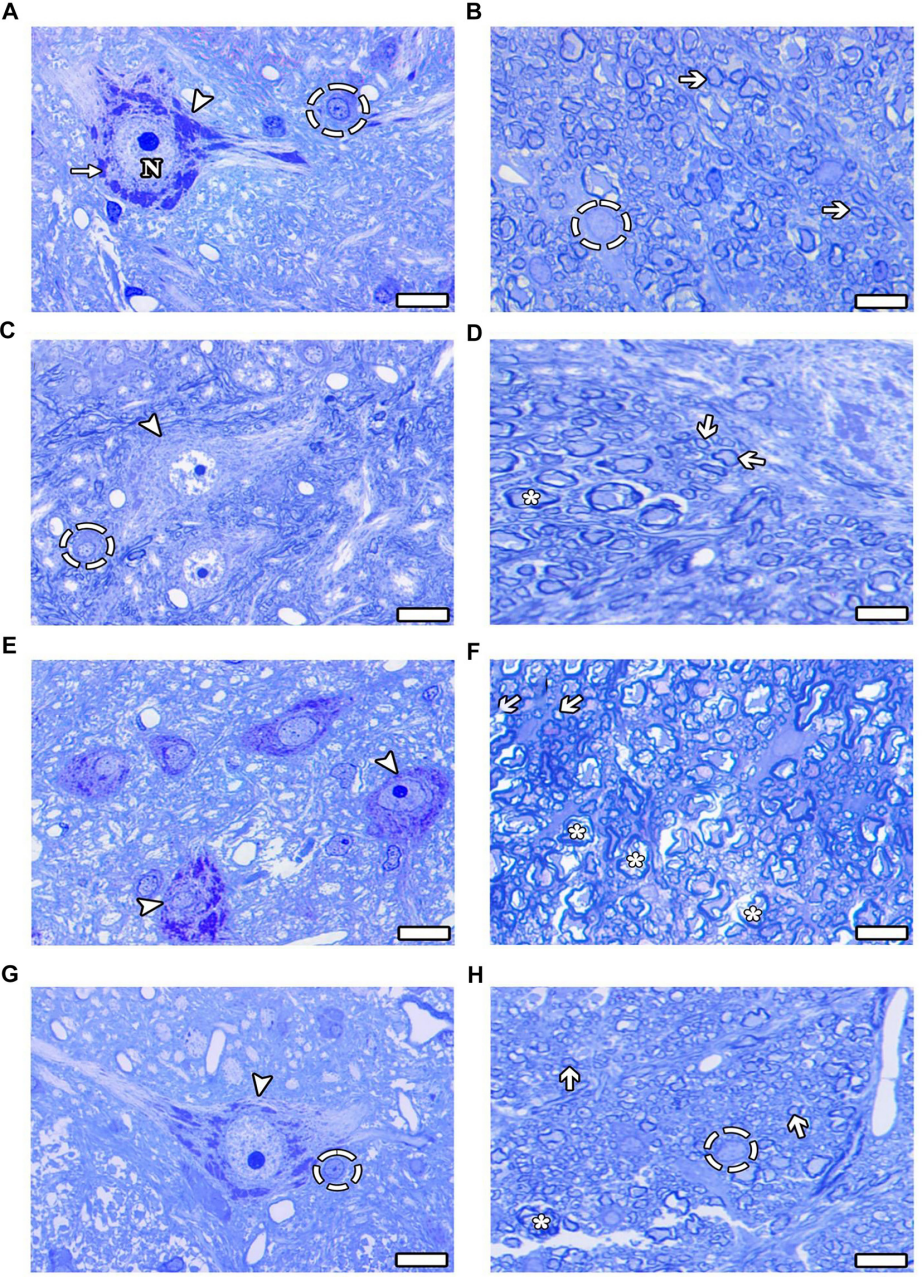
**(A–H) Light microscope images of the groups are presented in semi-thin sections.** (A and B) CONT group; (C and D) SHAM group; (E and F) Mel group; (G and H) ω3 group. Arrowhead: Healthy motor neuron; Thin arrow: Nissl body; Thick arrow: Healthy myelinated axon structure; Dashed circle: Healthy oligodendrocyte; (*): Degenerated myelinated axon; N: Nucleus. Scale bars: 10 µm-toluidine blue staining.

The morphologies of neurons, neuroglial cells, and axons were histopathologically examined and evaluated in semi-thin sections from all groups using light microscopy. Semi-thin sections stained with toluidine blue revealed that the motor neurons and neuroglial cells in the CONT (Figure 3A), SHAM (Figure 3C), Mel (Figure 3E), and ω3 groups (Figure 3G) exhibited normal morphology. The motor neurons located in the gray matter were oval-shaped, had well-defined borders, and displayed euchromatic nuclei, with neuroglial cells interspersed among them. Additionally, the intercellular spaces appeared normal, and the integrity of the surrounding tissue was preserved. Examination of the axonal structures in these groups indicated that the axons in the CONT (Figure 3B) and ω3 (Figure 3H) groups maintained healthy morphology, with well-preserved connective tissue adjacent to the axons. Although some axonal degeneration was observed in the SHAM group (Figure 3D), the density of healthy axons was notable. Furthermore, both myelinated axons with healthy and degenerated morphology were identified in the Mel-only group (Figure 3F).

**Figure 4.**
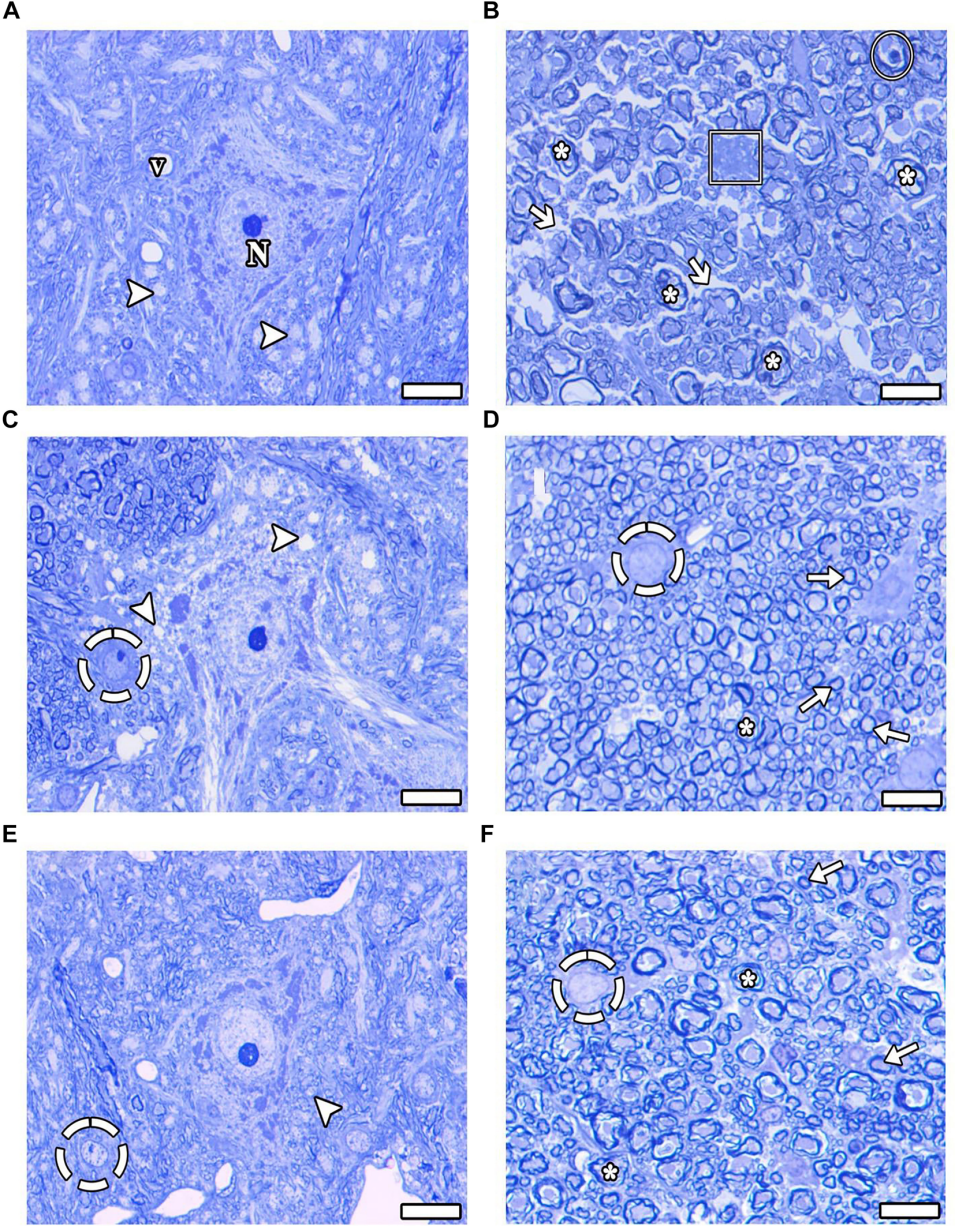
**(A–F) Light microscope images of the groups are presented in semi-thin sections.** (A and B) EMF group; (C and D) EMF-Mel group; (E and F) EMF-ω3 group. Arrowhead: Vacuolization around motor neurons; Thin arrow: Healthy myelinated axon structure; Thick arrow: Endoneurial disruption; Dashed circle: Healthy oligodendrocyte, (*): Degenerated myelinated axon; Circle: Schmidt-Lanterman cleft; Rectangle: Degenerated oligodendrocyte; N: Nucleus; v: Vessel. Scale bars: 10 µm-toluidine blue staining.

In the evaluation of semi-thin sections from the EMF (Figure 4A), EMF-Mel (Figure 4C), and EMF-ω3 (Figure 4E) groups, histopathological analysis of motor neuron morphology revealed vacuolization surrounding the motor neurons. Significant axonal degradation and oligodendrocyte degeneration were noted in the EMF group (Figure 4B). However, in the EMF-Mel (Figure 4D) and EMF-ω3 (Figure 4F) groups, the detrimental effects of EMF on cellular structures were mitigated, resulting in an abundance of healthy axons and oligodendrocytes that retained their morphology.

### Electron microscopic evaluation

**Figure 5.**
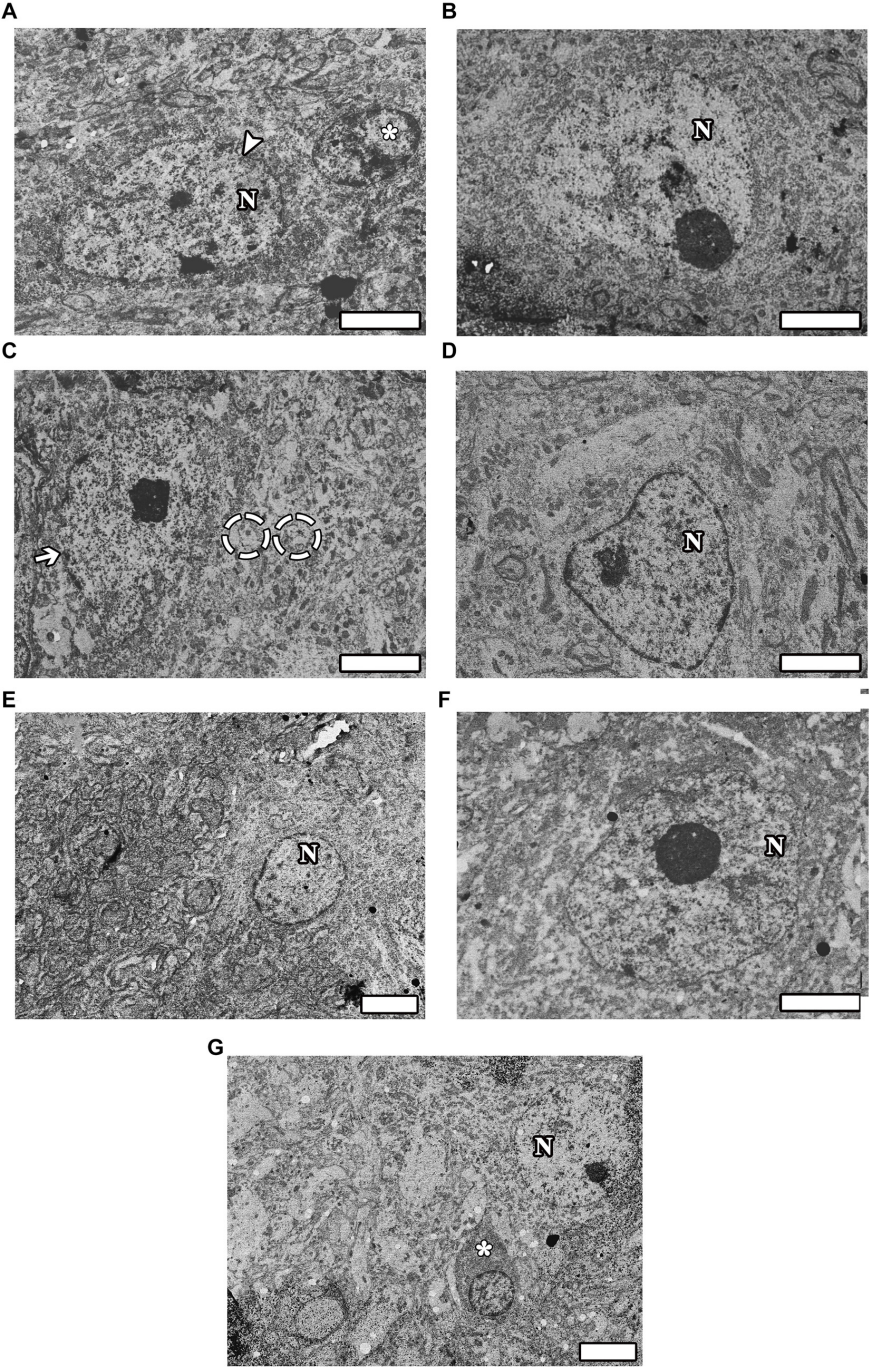
(A–G) Electron microscope images of the groups are seen taken from thin sections. (A) CONT group; (B) SHAM group; (C) EMF group; (D) EMF-Mel group; (E) EMF-ω3 group; (F) Mel group; (G) ω3 group. Arrowhead: Healthy motor neuron; Arrow: Neuron with disrupted membrane integrity; Dashed circle: Vacuolization around motor neuron; (*): Healthy oligodendrocyte; N: Nucleus. Scale bars: 1 µm.

The ultrastructure of motor neurons in the CONT group appeared healthy, with clearly defined boundaries of the nucleus and nucleolus. The glial cell nuclei exhibited distinct borders, and their nuclear structures were oval, stained with euchromatin (Figure 5A). The SHAM group also showed robust structures, with nuclei demonstrating euchromatic staining and clearly delineated boundaries of the nucleus and nucleolus (Figure 5B). In contrast, the ultrastructure of neurons in the EMF group revealed indistinct nuclear borders, with distorted neuron boundaries that had lost their spherical shape. Additionally, several instances of vacuolization were observed surrounding the motor neurons (Figure 5C). Conversely, the EMF-Mel (Figure 5D) and EMF-ω3 (Figure 5E) groups exhibited neurons with normal morphology, with the integrity of the nuclear membrane notably preserved. Moreover, the overall morphology of neurons in the Mel (Figure 5F) and ω3 groups (Figure 5G) appeared intact. The oligodendroglia in the ω3 group displayed a normal structure, characterized by distinctly visible nuclei and nucleoli.

### Stereological evaluation

**Figure 6.**
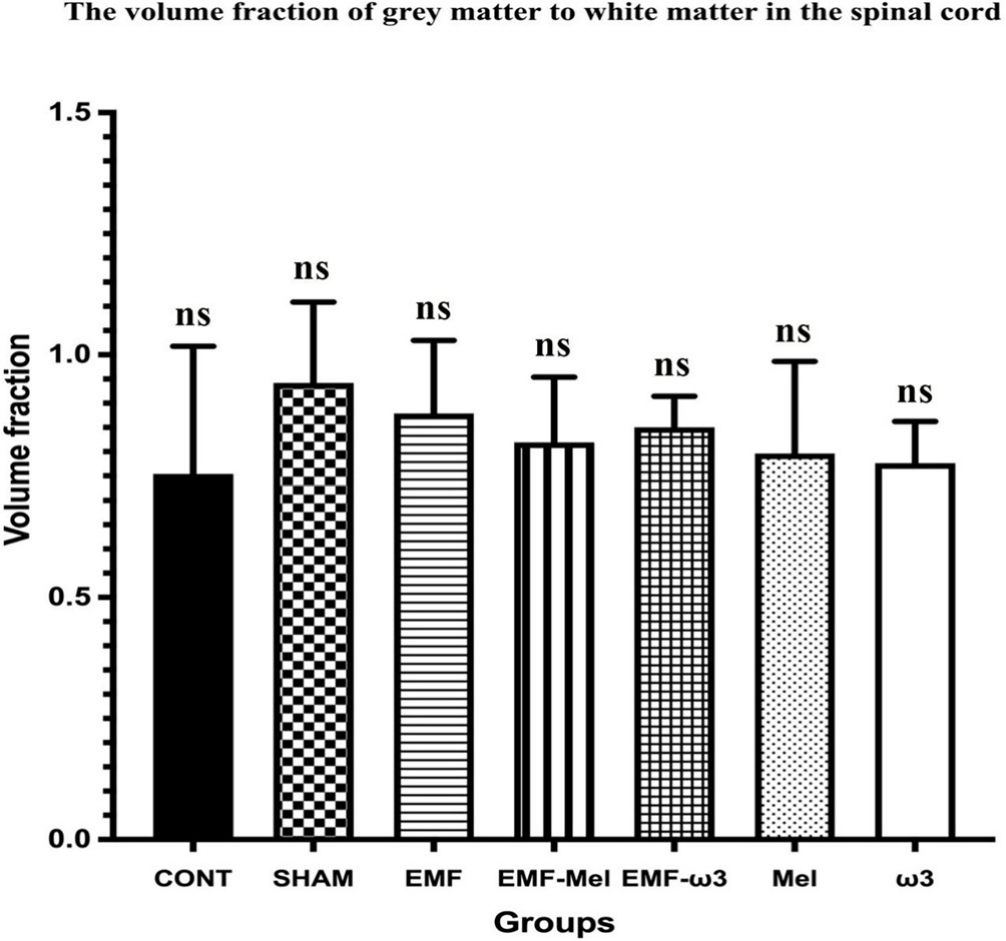
**Graph illustrating group differences in spinal cord GM/WM volume following 2-h EMF exposure.** No statistically significant differences were observed between the groups (*P* > 0.05); non-significance is indicated by ns.

**Figure 7.**
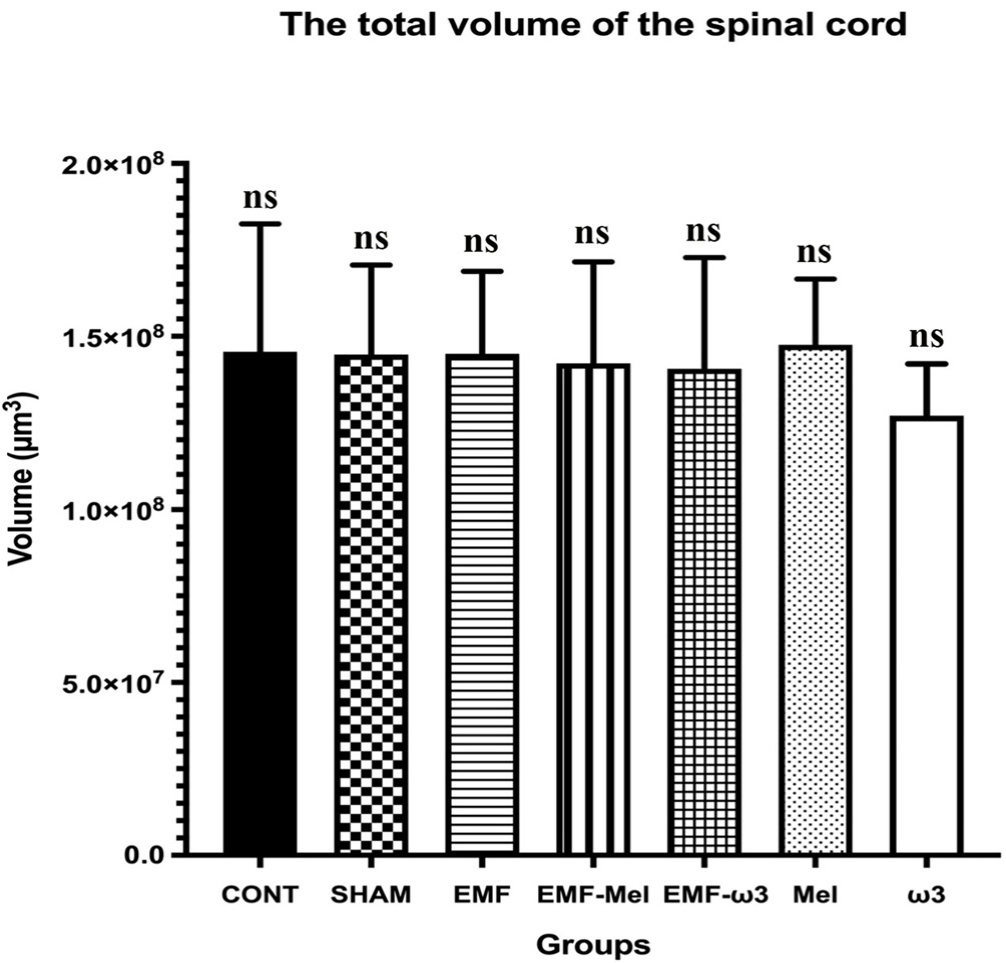
**Graph illustrating group differences in total spinal cord volume following 2-h EMF exposure.** No statistically significant differences were observed between the groups (*P* > 0.05); non-significance is indicated by ns.

**Figure 8.**
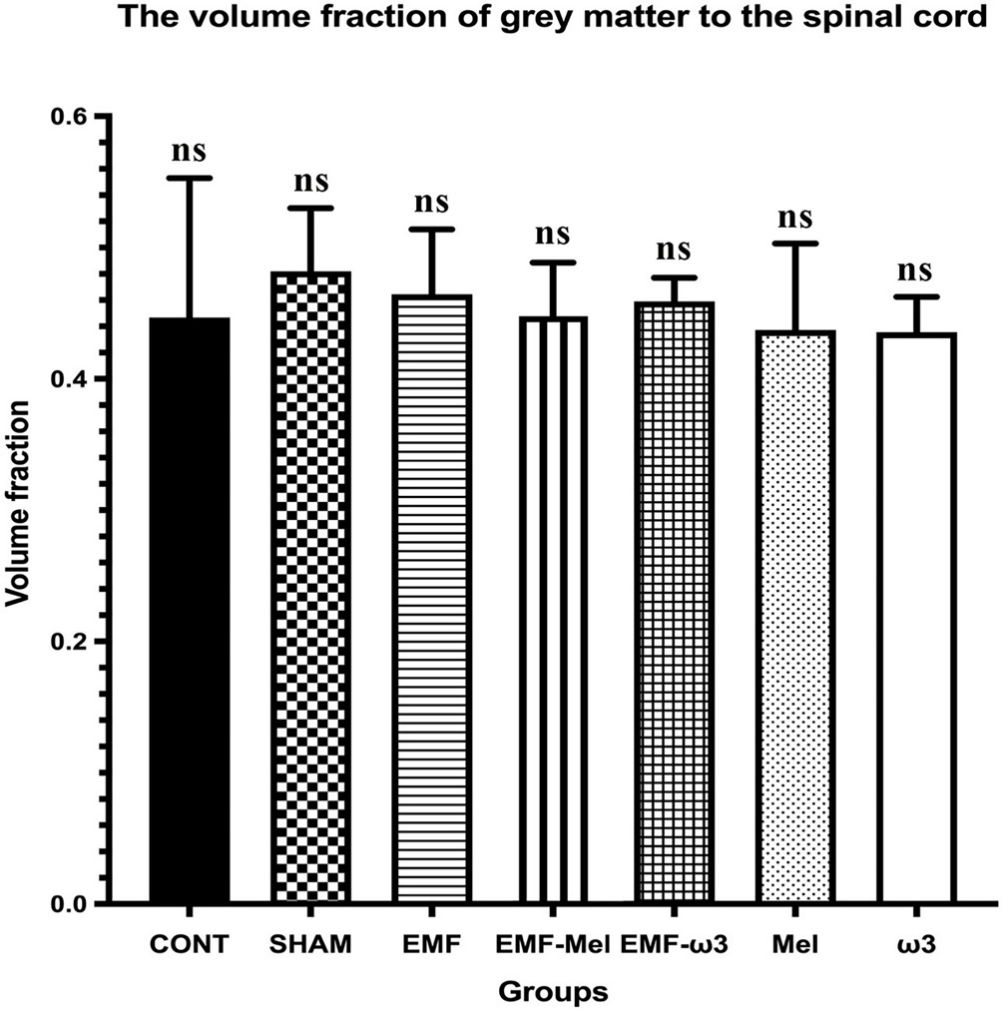
**Graph illustrating group differences in GM/total volume ratio following 2-h EMF exposure.** No statistically significant differences were observed between the groups (*P* > 0.05); non-significance is indicated by ns.

**Figure 9.**
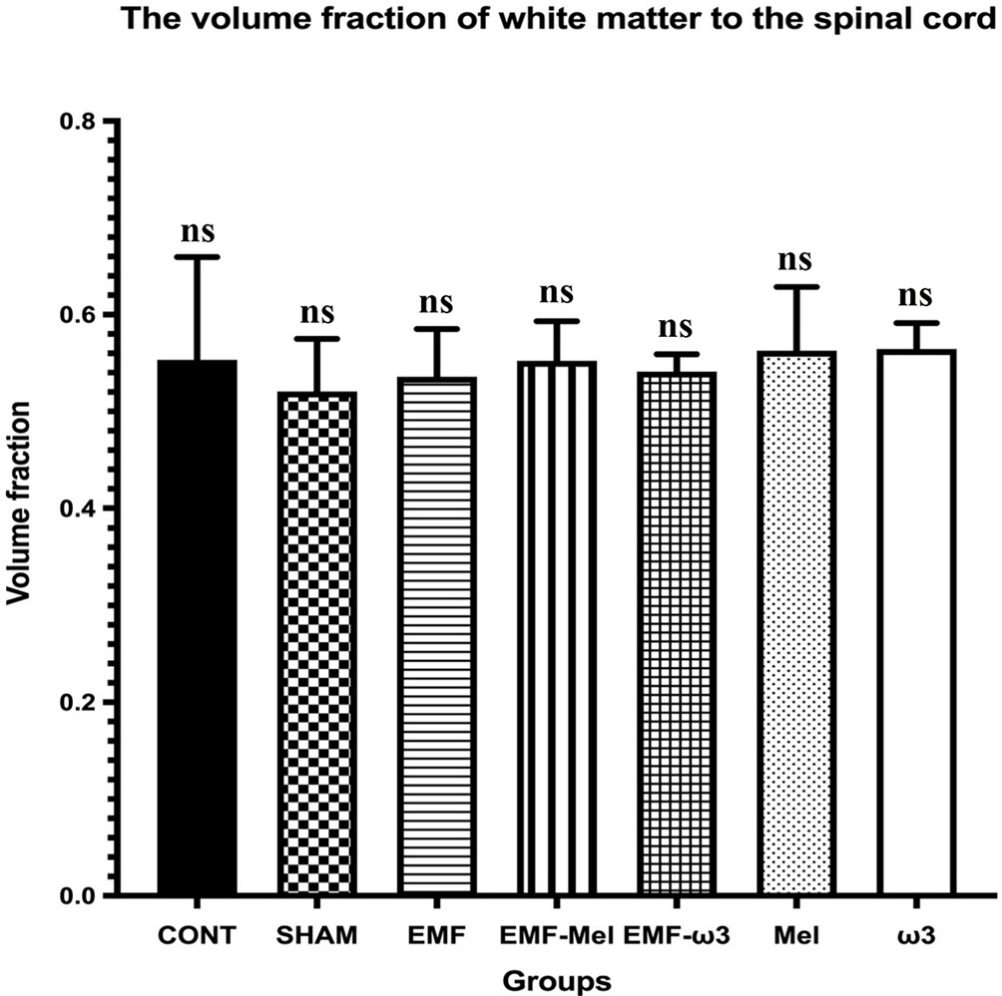
**Graph illustrating group differences in WM/total volume ratio following 2-h EMF exposure.** No statistically significant differences were observed between the groups (*P* > 0.05); non-significance is indicated by ns.

**Figure 10.**
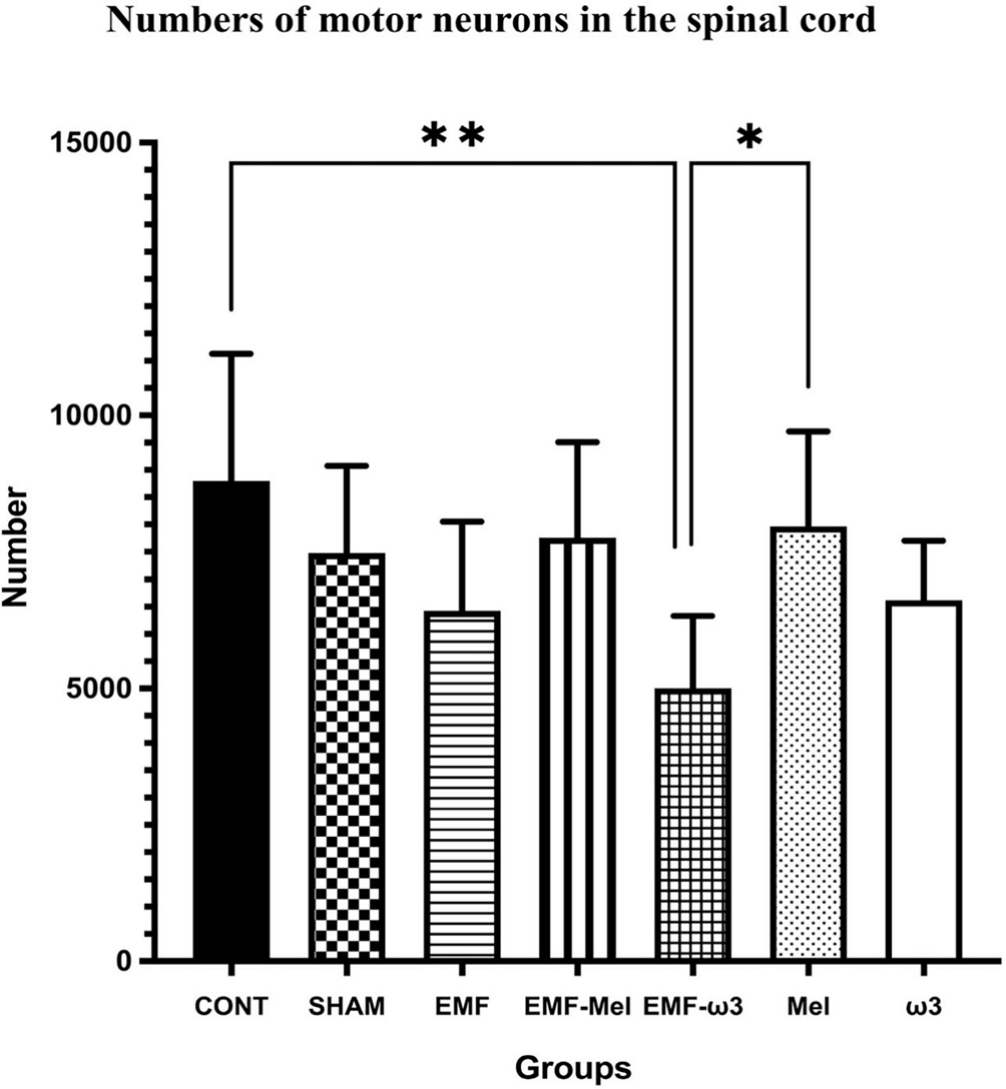
**Graph illustrating group differences in the number of motor neurons in the spinal cord following 2-h EMF exposure.** Statistically significant differences are indicated as follows: *P* < 0.05 (*), *P* < 0.01 (**).

In the relevant study, volume analyses were conducted using the Cavalieri principle, while motor neuron counts were obtained through the physical disector method—both of which are unbiased and reliable stereological techniques. In this context, no significant differences were observed among the groups regarding total volume (*P* > 0.05). Likewise, the volume fractions of GM to WM, GM to total volume, and WM to total volume did not exhibit significant differences between the groups (*P* > 0.05) (Figures 6–9). However, when assessing the number of motor neurons, a significant difference emerged among the groups (*P* < 0.01) (Figure 10). Specifically, the EMF-ω3 group displayed a significant decrease in motor neuron numbers compared to the CONT group (*P* < 0.01). Additionally, the EMF-ω3 group had a significantly lower count of motor neurons than the MEL group (*P* < 0.05).

## Discussion

High doses and prolonged exposure to EMF during pregnancy have been shown to induce cell death and inhibit the differentiation of neural stem cells in both prenatal and postnatal periods [21]. The detrimental effects of various teratogenic factors are more pronounced during the prenatal period compared to the postnatal period [22]. Notably, the embryo is particularly vulnerable to toxic agents during the first two weeks of the embryonic stage. Consequently, the teratogenic effects of EMF exposure during pregnancy have garnered significant research interest, especially given the widespread use of mobile phones. Considering the foetus’s sensitivity to maternal influences, it is crucial to investigate the impact of EMF exposure during the prenatal period on the central nervous system comprehensively. Recent studies have begun to examine the adverse effects of EMF exposure on the central nervous system, particularly due to the proximity of mobile phones to the head [6, 10, 23]. For instance, Yahyazadeh and Altunkaynak [24] demonstrated that exposure to a 900 MHz EMF adversely affected the spinal cord of rats. Similarly, İkinciKeleş [25] found that prenatal exposure to a 900 MHz EMF negatively impacted vertebral development, leading to pathological changes in rat pups. İkinciKeleş and BitergeSüt [5] reported that such prenatal exposure may induce structural changes in the rat spinal cord. Eid et al. [26] found that EMF exposure resulted in neural damage within the hippocampus and cerebellum. Additionally, Moussa et al. [27] reported significant structural alterations in the hippocampus of rats exposed to EMF, particularly in the CA3 region.

The primary mechanism by which EMF affects biological systems is through the generation of free radicals [28]. Organisms possess various enzymatic and non-enzymatic antioxidant defense systems to mitigate the effects of these harmful radicals. However, research indicates that conditions favoring the imbalance between free radicals and antioxidants can lead to increased oxidative stress. The use of antioxidant substances may alleviate this oxidative stress and reduce neuronal damage through inhibition [29, 30]. The present study investigated the protective effects of antioxidant agents such as Mel and ω3 on neurodegeneration occurring in the spinal cord of rats exposed to 900 MHz EMF in the prenatal period by means of stereological and histopathological methods. Mel is critically important for fetal development and promotes the proliferation of neural stem cells [31]. ErdemKoç et al. [32] utilized Mel to mitigate the effects of EMF on the developing brain and to protect pyramidal neurons in the hippocampal region, observing that maternal administration of Mel reduced the adverse effects of EMF on the fetal brain. Delen et al. [33] reported that RF radiation (RFR) exposure caused structural deformation and apoptosis in brain tissue, while Mel ameliorated these deleterious effects. Tüfekçi et al. [34] also noted that Mel exhibited a neuroprotective effect on the optic nerve of rats exposed to prenatal EMF. Omega-3 fatty acids play a significant role in nervous system development [35]. Bi et al. [36] demonstrated that ω3 supplementation reduced oxidative stress, apoptosis, and inflammatory markers in rats with spinal cord injuries. Multiple studies have reported positive effects of ω3 administration on neuronal morphology and counts [32, 37, 38].

Stereological methods enable the acquisition of unbiased and effective results, facilitating three-dimensional quantitative assessments of objects through their two-dimensional planar projections [39]. This study employed the physical disector method for quantifying spinal cord motor neurons in various groups, while the Cavalieri method estimated the volumes of relevant structures [39, 40]. We evaluated the effects of prenatal EMF exposure on spinal cord motor neurons and investigated the potential neuroprotective roles of Mel and ω3. Our findings indicated a significant decrease in the number of motor neurons in the groups exposed to EMF during the prenatal period, particularly in the EMF-ω3 group compared to both the control and Mel groups. This suggests that ω3 alone may be insufficient to counteract the neurotoxic effects of prenatal EMF exposure. As ω3 fatty acids are highly susceptible to oxidation, they may have exacerbated the oxidative effects stemming from EMF during prenatal neurodevelopment. A combined application of ω3 with other antioxidants may prove more effective in addressing the destructive effects of EMF. Additionally, factors such as bioavailability, duration of effect, and dosage of ω3 may influence its efficacy in treating EMF-induced damage. While we observed changes in the number of motor neurons due to EMF exposure, there were no significant differences in total spinal cord volume or the volumetric fractions of GM and WM among the groups. The cellular damage may not have been reflected in volumetric data. EMF-induced damage activates pathways related to oxidative stress and cell death at the cellular level, thus highlighting the importance of evaluating results at the neuronal level during the prenatal period. Although neuron loss occurs during this period, volumetric changes may be obscured by glial proliferation. Therefore, a comprehensive assessment of neurodevelopmental processes in EMF-induced damage should also consider the total number of oligodendrocytes and astrocytes to identify real changes in the brain. In alignment with the criteria established by Sugimoto et al. [20] for identifying damaged neurons, light microscopic examination of cresyl violet-stained sections revealed that cell boundaries in the EMF group were less distinct and that there were more neurons with narrow, dark-stained cytoplasm compared to the control group. The oxidative stress induced by EMF exposure may lead to apoptosis through the generation of apoptotic signals [41]. The presence of numerous darkly stained cells in the spinal cord suggests apoptotic processes following EMF exposure. Furthermore, Mel and ω3 administration appeared to mitigate the neurodegenerative effects of EMF in the spinal cord. The histopathological protection provided by Mel, known for its robust neuroprotective properties, was superior to that of ω3, potentially due to Mel’s ability to traverse physiological barriers owing to its amphiphilic nature. Examination of semi-thin sections stained with toluidine blue indicated that motor neurons and neuroglial cells in the control, sham, Mel, and ω3 groups exhibited normal morphology.

In the EMF, EMF-Mel, and EMF-ω3 groups, vacuolization surrounding the neurons was observed. Electron microscopic findings corroborated these observations and demonstrated that both Mel and ω3 mitigated this effect in nerve damage caused by EMF. Axonal structures across the groups revealed that axons in the control and ω3 groups maintained healthy morphology, while a small degree of axonal degeneration, which did not significantly affect overall morphology, was noted in the sham group. In the Mel group, numerous axons exhibited deteriorated morphology. Conversely, severe axonal degradation and oligodendrocyte degeneration were observed in the EMF group. In the EMF-Mel and EMF-ω3 groups, the detrimental effects of EMF appeared to be alleviated, with a substantial number of healthy axons and oligodendrocytes preserving their morphology. Thus, it can be inferred that Mel and ω3 may facilitate remyelination following toxic damage to oligodendrocytes in the central nervous system. Symptoms of electro-hypersensitivity resulting from excessive exposure to telecommunication devices may arise from damage to oligodendrocytes, which play a crucial role in myelin formation within the central nervous system [42]. The therapeutic effects of Mel and ω3, particularly on oligodendrocytes in relation to axonal damage caused by EMF, are noteworthy. Further studies elucidating the detailed mechanisms underlying the role of these neuroglial cells in EMF-induced nerve damage are warranted.

## Conclusion

This study has shown that ω3 treatment+exposure to 900 MHz EMF during the prenatal period creates toxic effect at the number of motor neuron in the spinal cord, but this side effect was not seen in terms of volume fraction. Our expectation in this experiment was to see toxic effects of EMF exposure on the motor neuron number and volume of spinal cord in the EMF group. Changes at the number of neuron level was observed only EMF-ω3 group. This may point that the developing nervous system is sensitive to a combined environmental stress factors such EMF and ω3. This discrepancy should be investigated. Further studies are needed to elucidate the mechanisms of a combined antioxidant with EMF exposure.
